# Glial Diversity and Evolution: Insights from Teleost Fish

**DOI:** 10.3390/brainsci15070743

**Published:** 2025-07-11

**Authors:** Carla Lucini, Claudia Gatta

**Affiliations:** Department of Veterinary Medicine, University of Naples Federico II, 80137 Naples, Italy; claudia.gatta@unina.it

**Keywords:** brain, fish, glia, microglia, macroglia, radial glial cells, neuroepithelial cells, ependymocytes, astrocytes, oligodendrocytes

## Abstract

Glial cells, once considered mere support for neurons, have emerged as key players in brain function across vertebrates. The historical study of glia dates to the 19th century with the identification of ependymal cells and astrocytes, followed by the discovery of oligodendrocytes and microglia. While neurocentric perspectives overlooked glial functions, recent research highlights their essential roles in neurodevelopment, synapse regulation, brain homeostasis, and neuroimmune responses. In teleost fish, a group comprising over 32,000 species, glial cells exhibit unique properties compared to their mammalian counterparts. Thus, the aim of this review is synthesizing the current literature on fish glial cells, emphasizing their evolutionary significance, diversity, and potential as models for understanding vertebrate neurobiology. Microglia originate from both yolk sac cells and hematopoietic stem cells, forming distinct populations with specialized functions in the adult brain. Neural stem cells, including radial glial cells (RGCs) and neuroepithelial cells, remain active throughout life, supporting continuous neuro- and gliogenesis, a phenomenon far more extensive than in mammals. Ependymocytes line brain ventricles and show structural variability, with some resembling quiescent progenitor cells. Astrocytes are largely absent in most fish species. However, zebrafish exhibit astrocyte-like glial cells which show some structural and functional features in common with mammalian astrocytes. Oligodendrocytes share conserved mechanisms with mammals in myelination and axon insulation.

## 1. Historical Background

In 1836, Purkinje visualized the lining of the inner cavities of the brain and described striking cilia on its surface. This lining was called the “ependyma” and its composing cells were termed ependymal cells. Rudolf Virchow, in his 1858 book *Cellular Pathology*, first reported the term “neuroglia” regarding the previously described “connective tissue”. Subsequently, astrocytes were the first distinguished group of glial cells, and only at the beginning of the XXth century were oligodendrocytes and microglia added. In 1950, myelin (previously described by Virchow) was recognized as a specialization of Schwann cells [1]. In addition to the description of the four major groups of adult mammal glia, studies on the brains of embryos at the end of the 19th century by Kölliker, His, and Golgi led to the discovery of radial glial cells [2].

However, the initial research on glial cells subsequently declined after neurons attracted increased attention after the discovery of action potentials, synapses, and neurotransmitters. Consequently, the prevailing vision of brain activity became neurocentric. Nevertheless, more recent evolutionary studies on organisms ranging from invertebrates to rodents and humans reported that the glia-to-neuron ratio increases with nervous system complexity and glial cells appear to be functionally more and more diversified [3,4]. During development, glial cells aid axon guidance, synapse formation, and pruning. In adults, they regulate brain maintenance, learning, memory, and injury response [5,6].

From an evolutionary point of view, it soon became clear that the classical morphological division of brain glia into astrocytes, ependymal cells, oligodendrocytes, and microglia can only be applied to mammals. However, despite the differences that exist, many common functions and markers are present in the glia of vertebrates and of invertebrates [7].

In the following paragraphs, the present review attempts to define, in light of the current literature, the characteristics and the roles of glia in fish, a group of vertebrates which contains more than half of vertebrate animal species. Teleost fish, a group of bone fish, is composed of more 32,000 species. In addition to having great economic importance as a human food source, several teleost species (i.e., goldfish, medaka, zebrafish) are important animal models for scientific purposes, being adopted in numerous laboratories all over the world.

## 2. The Teleost Fish Brain: An Overview

The teleost fish brain can be subdivided into the telencephalon and diencephalon (forebrain), mesencephalon (midbrain), and rhombencephalon (hindbrain). Orally, the telencephalon shows olfactory bulbs and the proper telencephalon, which was once considered to be simply an olfactory center and subsequently considered to have several associative properties. The diencephalon, ventrally extending below the mesencephalon, is composed of five major regions: the epithalamus, dorsal thalamus, ventral thalamus, posterior tuberculum, and hypothalamus. In general, the teleost diencephalon processes sensory information and regulates endocrine functions and homeostasis. Dorsally, the mesencephalon shows the optic tectum, a large, paired region with a complex layered cortex involved in processing optic stimuli and integrating information from the forebrain, the hind brain, and the tegmentum, the ventral part of the mesencephalon. The tegmentum processes auditory signals (tori longitudinales) and includes the oculomotor nuclei. The rhombencephalon includes two regions, the cerebellum and medulla oblongata. The cerebellum is composed of valvula, the part which extends below the optic tectum into the mesencephalic ventricle, and dorsally, it is composed of the corpus and two paired eminantiae granulares. The cerebellum has a three-layered cortex highly similar to the mammalian one and has a crucial role in fine-tuning motor control, coordination, and learning. The medulla oblongata contains the sensory and motor nuclei of V–X, thus integrating sensorimotor coordination. It continues, after passing the cranial foramen magnum, as the medulla spinalis [8,9].

## 3. Microglia

Microglia, a group of small resident glial cells in the central nervous system (CNS), are defined as resident macrophages of the brain due to their phagocytic roles in the CNS. In fish, microglia have been described in different fish species [10,11], mostly zebrafish [12,13,14,15,16]. Despite the fact that most of the morphofunctional characteristics of microglia are majorly conserved in vertebrates [17], fish microglia appear particularly able to participate in the repair and regeneration of structures produced after an injury, as well as in atrophy and degeneration [18]. Microglial cells may represent 5–20% of the resident glial cells in the brain of adult fish, although these percentages may vary depending on the fish species, and one third of them may be found in the optic nerve. Nevertheless, the number of activated microglia present in a particular area has recently been found to be directly related to the response to different types of damage and their possible role in regeneration/degeneration [19].

In early zebrafish embryos, microglia derive from the yolk sac [12], like in mammals. However, differently from mammals, a second wave of microglial colonization, derived from hematopoietic stem cells, contributes to the adult pool [20,21]. In adult zebrafish, three distinct microglial populations have been identified. In the midbrain, there is a predominant population that is broadly distributed, amoeboid in shape, highly mobile, and phagocytotic, and another population with ramified protrusions but limited mobility and phagocytosis capability [22]. Also, in the telencephalon, a further macrophagic population has recently been described [23].

## 4. Neural Stem Cells, Neuroepithelial Cells, and Radial Glial Cells

Neural stem cells (NSCs) are traditionally defined as multipotent progenitors able to self-renew throughout their lifetime to generate diversity in neurons and glial cells [24]. During development, NSCs mix expansion—through symmetric division—with differentiation—through asymmetric division [25]—to ensure that the correct amount of progeny are generated while preserving their pool. In adults, NSCs are also present and active, but their extent varies between species. In adult rodents, NSCs are present only in restricted locations: the subependymal zone of the lateral ventricle and the subgranular zone of the dentate gyrus of the hippocampus. Particularly in humans, adult neurogenesis seems to decline with age [26], although contrasting results could be ascribed to methodologies and neural stem heterogeneity [27,28]. Conversely, in most teleost fish, the brain keeps growing along with the skull, and NSCs are found in extensive locations, providing constant neuro/gliogenesis [29]. Briefly, since the complex topic of adult neurogenesis in fish is beyond the scope of this review, only data regarding the zebrafish, whose adult neurogenesis is the most studied, is provided below.

In zebrafish, using classical tracing methods, 16 proliferation domains were seen to be present across all brain subdivisions [30,31,32,33]. Particularly in the telencephalon, adult neurogenesis has been studied for longer, due to its territories that are retained homologously to the two main neurogenic niches of adult rodents: the dorsal–lateral zone of the telencephalon, homologous to the medial pallium (hippocampus) of rodents [34], and the periventricular zone of the ventral telencephalon in zebrafish, homologous to the rodent subependymal zone of the lateral ventricle [31]. In the dorsal telencephalon, NSCs appear as radial glia cells (RGCs), organized in a continuous epithelial-like sheet, with basal processes contacting blood vessels at the pial surface, and with apical processes in the ventricular zone in contact with the cerebrospinal fluid [35] and expressing components of adherens and tight junction complexes [36]. Dorsal telencephalic RGCs show some astroglial and ependymal markers, such as glial fibrillary acid protein (GFAP), S100b, brain lipid-binding protein, Fabp7a, Sox2, Her4.1, Hey1, and Nestin. Heterogeneity between these cells has also been proven, classifying them as predominant type I, in a quiescent state, and type II, a few actively proliferating cells identified by PCNA or MCM2/5 expression or via prevalent Notch signaling [31,37,38,39]. Also, using single-cell transcriptomic analysis (sc-RNAseq), the heterogeneity of RGCs has been described [40,41], as numerically predominant quiescent and less numerous proliferative RGCs are spatially patterned [23]. The quiescent RGCs, with lineage progression, downregulate the expression of astroglial and ependymal markers, generating neural progenitors that undergo a limited number of divisions before delaminating from the ventricular surface, giving rise to Hu-positive neurons in the parenchyma below [31,42,43]. These predominant quiescent RGCs pool in zebrafish, which is initiated in zebrafish between 7 and 10 days postfertilization, maintaining the adult NSC pool over the lifespan [44]. On the contrary, in mice, the continuous proliferation by NSCs, required to generate the CNS during development, leads to their exhaustion, despite their capacity for asymmetric self-renewing divisions.

In the ventral telencephalon, NSCs are highly proliferative neuroepithelial cells (NECs) that express the intermediate filament nestin but are negative for GFAP expression [31,45]. They give rise to neurons of the ventral telencephalon, as well as to migratory progenitors that transit tangentially alongside blood vessels to the olfactory bulb, where they produce GABAergic and dopaminergic neurons [31,32,35]. In the hypothalamus, the paraventricular organ exhibits numerous proliferating RGCs along the ventricular surface that extend towards the ventricle’s cytoplasmic processes [46]. In the optic tectum, the caudal margin of the inner layer (periventricular gray zone) shows fast proliferating cells with neuroepithelial characteristics, such as apical–basal polarity and the presence of occludent junction markers, as well as the absence of glial markers. They generate both glial and neuronal lineages [47]. In the cerebellum, neuroepithelial cells continuously give rise to granule cells in adult and aging zebrafish and are the predominant stem cell type supporting cerebellar regeneration after injury. Instead, RGCs play a minor role in adult cerebellar neurogenesis and in recovery after injury [48,49].

## 5. Ependymocytes

In mammals, the surface of the brain’s ventricles and the canal of the spinal cord are layered by ependymocytes, ciliated cells that regulate the cerebrospinal fluid flow, which creates a sort of epithelial layer or “ependyma” [50]. Looking at the optic tectum of sand bass, Kruger and Maxwell [51] conducted light and electronic microscopical studies and described ependymocytes lining the brain ventricles with cilia until reaching the cerebrospinal fluid and long fibrillar processes up to the pial surface. In goldfish, Stevenson and Yoon [52] reported ependymocytes with cytoplasm containing prominent bundles of intermediate filaments but not microtubules. Their soma lies at or near the ventricles.

Subsequently, the presence of vimentin and GFAP was considered to be a marker of ependymocytes. The fish equivalent of mammalian vimentin was identified in the optic nerve of cichlid fish *Tilapia nilotica* in [53]. GFAP was reported in the ependymocytes of goldfish brain [54,55] and Iberian barb brain [56]. However, GFAP- and/or vimentin-containing glia might be differentiated, secondarily specialized, astroglia-like cells [57,58]. GFAP is generally considered a marker for astrocytes in mammals [59,60], and vimentin filaments are a class of intermediate filaments that form the cytoskeleton in eukaryotic cells, thus making them not exclusive for glial cells [61]. As a consequence, non-GFAP and non-vimentin-positive ependymal cells have been proposed to represent “authentic” ependymocytes [57].

In zebrafish embryos, ependymocytes show solitary cilia that move in a rotational manner; successively, as the brain and its ventricles expand, multiciliated cells similar to mammalian ependymocytes progressively appear in the forebrain (on the tela choroidea of the telencephalic ventricle, choroid plexus, and along the parenchymal surface in the midline). Some multiciliated ependymal cells, which are heterogeneous in their genetics and functional properties, result from single-cell transcriptomics and are genetically like progenitor quiescent cells [62].

## 6. Astrocytes

Astrocytes are the most abundant glial cell type in the mammalian CNS [63]. They have a branched morphology and exhibit fine cellular processes that interact closely with synapses, neuronal cell bodies, axons, blood vessels, and other glial cells in the CNS.

Fish astrocytes were first described using transmission electron microscopy [64]. Using scanning electron microscopy, Castejon and Caraballo [65] reported astrocytes in the cerebellum of a catfish. Immunocytochemical studies, using antibodies against GFAP, a known marker of astrocytes in the mammalian brain, have found a lack of astrocytes in fish brains [55,56,66,67,68]. Also, a large study based on the immunohistochemical staining of GFAP, mainly conducted in the telencephalon, tectum, and cerebellum of several actinopterygian fish species at different phylogenetical positions, demonstrated that, in Actinopterygii—in contrast to Chondrichthyes and Amniotes—true astrocytes (stellate-shaped extraependymal cells) did not appear during evolution [69]. However, Maggs and Scholes [70] depicted glial cells as “reticular astrocytes”; the former do not display GFAP IR and form a perinodal association, as mammalian astrocytes usually do, with the optic nerve, strictly linked by tight junctions. Furthermore, in the spinal cord and several areas of the brain of zebrafish, star-shaped cells without axon-like structures, containing the GFAP marker and contacting veins, were indicated as astrocytes [71].

In the spinal cord of zebrafish, Chen et al. [72], by means of time-lapse in vivo confocal microscopy, showed that RGCs begin to transform from RGCs into astrocyte-like cells at 2 d postfertilization, displaying dynamic process elaboration over the course of development. Also, in both the brain and spinal cord of zebrafish, by generating a stable transgenic line that labels the membrane and nuclei of all glutamate aspartate transporters, this cell population showed dense cellular processes that are remarkably similar to those of mammalian astrocytes. These cells exhibit several additional defining characteristics of mammalian astrocytes, including a close association with synapses and astrocyte tiling behavior.

In the zebrafish telencephalon, among quiescent RGCs, several clusters were identified, driven by quiescence duration and depth. Among the genes associated with deeper quiescence in zebrafish RGCs, several were also expressed in both quiescent RGCs and astrocytes in mice. This revealed that the similarity with mammalian astrocytes in zebrafish RGCs increases with the estimated quiescence depth. Gene ontology analysis of the genes of some clusters of quiescent RGCs demonstrated genes encoding proteins involved in astrocytic support functions such as neurotransmitter synthesis and recapture, metabolic support, the maintenance of ionic balance, and the modulation of ECM properties [23].

## 7. Oligodendrocytes

Oligodendrocytes are cells that, by wrapping CNS axons with thin processes, create the myelin sheath, which allows the saltatory conduction of action potentials. Myelin is present in all vertebrate gnathostomes. In addition, oligodendrocytes provide trophic and metabolic support to neurons [73].

In fish, oligodendrocytes were first described in the brain of the rainbow trout by means of NADPH-d histochemistry. They were described as having richly branched processes associated with myelin sheaths. Also, they were found to be heterogeneous regarding the cell size, shape, and number of processes, showing a positive correlation between the size of axons and that of the associated oligodendrocytes [74]. Ultrastructurally, oligodendrocytes show few thin processes, and contain long microtubules, numerous free ribosomes, and a cytoplasmic matrix that is clearly denser than that of other glial cell types and neurons; thus, they are fundamentally comparable to human oligodendrocytes[75,76,77].

Regarding their origins, NSCs originate oligodendrocyte progenitor cells (OPCs) that differentiate into pre-myelinating oligodendrocytes, which subsequently mature into fully functional oligodendrocytes. The differentiation of OPCs is stimulated and regulated by neuronal activity and contributes to neuroplasticity, learning, and memory. All these processes are conserved from zebrafish to humans [78].

## 8. Conclusions

The typical classification of adult mammalian glia, dividing it into microglia, astrocytes, oligodendrocytes, and ependymoglia, is not automatically applicable to fish, primarily due to the massive presence of NSCs in the brains of adult fish, which allow for continuous adult neurogenesis and, consequently, a high regenerative potential. Moreover, in fish, there do not appear to be cells morphologically comparable to astrocytes. However, among the quiescent-type RGCs and ependymocytes, some can, based on markers and functions, be compared to astrocytes. These distinctive features of fish glial cells should therefore be taken into account in studies on the nervous system and its pathologies that employ fish as experimental models.

More speculative considerations might suggest that certain functional properties of glial cells could precede morphological specialization in the course of evolution.The more complex neuronal networks of mammals, despite sharing numerous cellular and neurostructural similarities with those of teleost fish, develop within a highly specialized glial environmental context. It would not be entirely inappropriate to attribute a significant role in this evolutionary trajectory to the adaptive morphophysiology of glial cells.

## Data Availability

No new data were created.
